# Clinical and Economic Evidence Supporting the Value of Fluorescence Imaging of Bacteria in Wound Care

**DOI:** 10.3390/jmahp13040048

**Published:** 2025-09-26

**Authors:** Jonathan Johnson, Gregory Bohn

**Affiliations:** 1 Comprehensive Wound Care Services, Washington, DC 20037, USA; 2 Lourdes Health, Pasco, WA 99301, USA

**Keywords:** bacteria, cost savings, expenditures, fluorescence imaging, infection, point of care, wounds

## Abstract

Wound infection significantly hinders the healing process. Clinical signs and symptoms (CSS) of infection are used to assess the presence of infection and guide whether to intervene. However, CSS may not be dependable, lacking sensitivity and specificity, and may not accurately reflect bacterial load. The interpretation of CSS can be subjective and can vary between clinicians since they depend on patient characteristics, type of wound, and stage of infection. In addition, conditions such as peripheral vascular disease or diabetes can mask the signs and symptoms of infection. Inaccurate or late diagnosis of infected wounds can be costly to the patient and to healthcare systems. Fluorescence imaging (FLI) provides a safe, objective, highly sensitive approach to detect clinically significant bacterial levels in wounds. This information allows individualized treatment plans and a way to monitor bacterial burden and wound healing longitudinally. This publication reviews the evidence for point-of-care FLI as a means of improving wound identification with a high bacterial burden and the clinical and healthcare economic benefits of earlier and more accurate detection of bacteria.

## 1. Introduction

**Figure 1:**
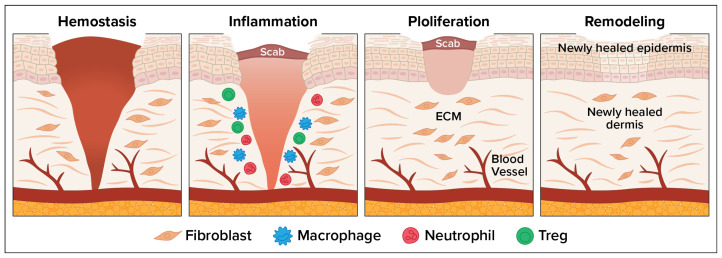
Four Phases of the Wound Healing Process.

The wound healing process consists of four overlapping but distinct phases: (1) hemostasis, which involves vascular constriction and platelet aggregation leading to clot formation; (2) inflammation, allowing pathogens and debris to be removed; (3) tissue regeneration, involving fibroblast proliferation, angiogenesis, and keratinocyte migration to re-epithelialize the wound; and (4) remodeling or maturation when the tissue is reorganized and strengthened [1]. Figure 1 depicts the wound healing process. Wound infection disrupts the normal healing process, often leading to chronic wounds, delayed closure, or systemic complications. Bacterial colonization leads to biofilm formation. Infected wounds show excessive neutrophilic infiltration, increased reactive oxygen species, protease release, and cytokine dysregulation. Chronic inflammation leads to tissue necrosis, fibrosis, and persistent non-healing states [2]. 

Analytical tools used to assess infected wounds include subjective assessment of clinical signs and symptoms (CSS), microbiological analysis using swab culture, tissue biopsy, or quantitative culture. Other technologies used include histology and immunohistochemistry; assays to detect molecular or biochemical markers; fluorescence imaging (FLI); biosensors to detect pH, temperature, oxygen, or microbial toxins in real-time; smart dressings to monitor wounds dynamically; and biofilm detection assays [3]. Table 1 summarizes the currently available tools for bacterial detection and their key strengths and limitations.

Infections can develop in any wound, but diabetic foot ulcers (DFUs), venous leg ulcers, pressure injuries, wounds associated with surgical sites, and burns are at higher risk for infection. Bacteria can extend outside the wound bed, remain in the wound but penetrate deeply into the tissue and persist even after vigorous debridement, or extend invasively beyond the margins of the wound bed and surrounding wound area. Many wounds with high bacterial burdens do not result in known CSS associated with infection [4,5]. CSS are inherently unreliable in patients with typical chronic wounds [6]. New technology using FLI can be used to detect bacterial burden in wounds. This approach relies on the presence and excitation of endogenous fluorophores such as porphyrins or pyoverdines, produced by bacteria, which then emit visible red or cyan fluorescence when exposed to specific light wavelengths, typically in the blue/violet range, around 405 nm. An imaging device captures real-time images of the wound showing color-highlighted areas with bacterial load. This can guide clinicians in debridement or targeted antimicrobial use [7].

## 2. Clinical Concerns and Challenges with Wound Infection

Infected wounds afflict millions of people globally. High levels of bacteria in wounds can impede wound healing, cause pain and personal suffering, and increase the cost of patient care [8,9,10,11]. If these bacterially burdened wounds are not properly cleaned and treated, they can lead to cellulitis and more invasive infections, spreading to deeper tissues or the bloodstream, causing more serious complications including bacteremia, endocarditis, osteomyelitis, or necrotizing fasciitis. Recurrent episodes can lead to more invasive infection or other costly and serious consequences such as hospitalization and amputation [8,11].

**Figure 2:**
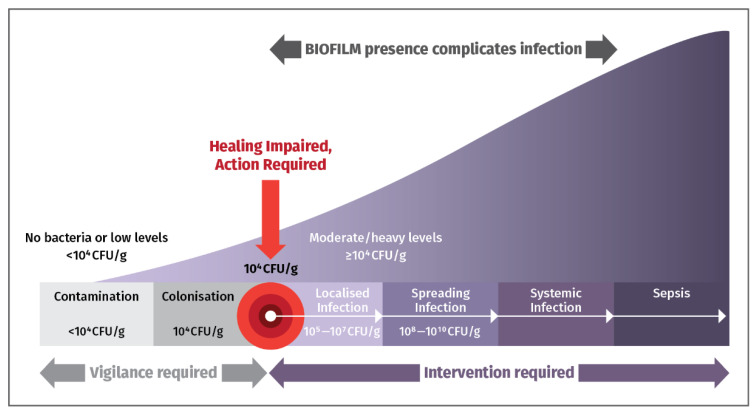
The Wound Infection Continuum. When wound bacterial loads exceed 10^4^ colony-forming units per gram (CFU/g), intervention is needed to handle biofilm and deter serious infection from occurring [12].

Figure 2 highlights the importance of promptly detecting and eliminating bacterial loads and biofilm in chronic wounds to prevent complications, and hospitalizations. Early detection allows sensible use of antimicrobial drugs to prevent unpleasant sequelae. Early intervention is essential to counter presence of bacteria [12].

Biofilms are a major impediment to wound healing, particularly in chronic wounds. These complex microbial communities, encased in a self-produced extracellular matrix, promote antimicrobial resistance, suppress immune response, sustain a pro-inflammatory environment, and impede tissue regeneration [13].

Chronic wounds can be treated with advanced technology/therapies such as autologous grafts, cellular and tissue-based products (CTPs)/skin substitutes, negative pressure wound therapy (NPWT), or hyperbaric oxygen therapy (HBOT). Results from costly therapies with high co-pays from patients often fail or are suboptimal due to undetected bacterial loads in the wound [14,15].

Surgical wound infections can lead to poor wound healing, chronic pain, hospitalizations, and increased risk of complications [16] and can significantly increase healthcare costs [17]. In addition, the use of antibiotics to treat these surgical wound infections contributes to the growing problem of antibiotic resistance, making it more difficult to treat future infections [18]. Prevalence of these wound infections is often underestimated because they occur after patients are discharged [19].

For burn wounds, infection is the most common life-threatening complication, with up to 75% mortality among those with burns. Early and accurate identification of infection in these patients is important to prevent the harmful sequelae [20,21]. Diagnosing burn wound infections requires evaluating clinical signs like erythema, induration, warmth, and tenderness, alongside objective measures like quantitative cultures and tissue biopsies, with the American Burn Association providing specific criteria for burn-related sepsis [22]. Identification of infections is necessary to initiate early removal of bacteria and allow for successful reconstructive surgery with CTPs and skin grafts [14,15].

In general, both wound measurement and assessing bacterial status are important for monitoring progression, informing treatment, and predicting wound healing. Hard-to-heal wounds are associated with <25% wound area reductions after 4 weeks of treatment and the presence of bacteria at loads of ≥10^4^ CFU/g [23].

## 3. Clinical Challenges with the Detection of Bacteria in Wounds

CSS are subjective, have limited predictive value and low sensitivity for high levels of bacteria [4,8]. There are three common techniques to detect infection that are used in addition to visual assessment of CSS: swab culture, deep-tissue biopsy, and needle aspiration. The presence of bacteria can be confirmed using wound sampling, culturing, and semi-quantitative microbiological analysis [24,25]. Swab culture is the preferred approach because it is simple, practical, and non-invasive. However, tissue sampling in chronic, hard-to-heal wounds show variability and can yield unreliable microbiological and sensitivity reports which then result in selection of antimicrobial therapies that are not appropriate [25]. Discrepancies exist between the gold standard biopsy and swabs. Swabs may not detect all types of bacteria and are unable to evaluate presence of deep-tissue microbial levels [26]. In addition, microbiological analysis is expensive, takes a few days, and can produce inaccurate results. The approach is subjective and unable to detect clinically significant levels of bacteria in asymptomatic patients [27].

In contrast to chronic infections typically involving multiple pathogens and biofilms, which can show delayed healing, wound breakdown, and less obvious signs, acute infections are often caused by a single organism, have clear signs like pain, redness, and purulent drainage, and are therefore often easier to detect [3].

## 4. Cost and Economic Burden of Wound Care Needed When Wounds Are Infected

An analysis of Medicare beneficiaries showed a 4% prevalence of surgical wound infections and 3.4% diabetic wound infections with a total mid-range estimate Medicare spend of $13.1 billion and $6.9 billion, respectively [28]. A 2019 publication provided a conservative estimate of annual wound care of greater than $50 billion in the US and £5 billion in the UK [28,29]. Risk of severe complications increases along with cost of care the longer a wound remains open [30]. Costs for wound care are primarily driven by spending by ineffective and/or reactive management of infection. Healthcare costs increase due to prolonged hospital stays, expanded resource requirements, and the need for advanced treatments in patients with infected wounds [31]. Unplanned readmissions, re-operations, extended antibiotic therapy, additional medications, dressings, and specialist consultations result in additional expenses and lead to poorer long-term patient outcomes [31]. Chronic, nonhealing wounds impact 8.2 billion Medicare beneficiaries annually, with the estimated annual cost of care, including the management of infection, between 28.1 and 96.8 billion US dollars [28].

**Cost burden from DFU infections.** Nearly 500 million people worldwide have been diagnosed with diabetes, and an estimated 1 in 3 will develop a DFU. Infections contribute to poor rates of DFU healing, as well as higher costs of patient care, and more minor lower extremity amputations [8]. Infection is the driving force for hospital admission and duration of hospitalization [32]. In a retrospective analysis of data from the Healthcare Cost and Utilization Project Nationwide Inpatient Sample from more than 962,000-foot ulcer admissions in the US, infection was the basis for 30.8% of DFU hospital admissions [32]. Almost 33% of costs for diabetes have been due to DFU treatment, and the majority of these were a result of hospital inpatient admissions [32]. Hospital costs for a DFU admission were 38.6% higher if the DFU was infected foot compared with all other causes [32]. Inaccurate or delayed diagnosis of infection contributed to some of the daily amputations related to DFUs. Undertreatment and overtreatment of wound infection can lead to suboptimal wound care, inflated costs, and antibiotic misuse [4,33,34].

Infection, in addition to ischemia from peripheral arterial disease, results in longer hospitalization (*p* < 0.01), longer healing time (*p* = 0.04) and higher hospitalization costs (*p* = 0.01) [35]. Infected ulcers are preventable. Minimizing infection can reduce hospital and treatment costs [32,35].

**Direct and indirect healthcare costs from surgical site infections (SSIs).** The definition of SSI according to the Center for Disease Control and Prevention (CDC) and the European Centre for Disease Prevention and Control is a postoperative infection within 30 days of surgery or within 1 year of a permanent implant [31]. In a population-based retrospective study, overall, the 30-day SSI rate was 13.5%, with more than 50% diagnosed after discharge. After hospital discharge, SSIs are associated with more emergency department visits, more hospital re-admissions, longer hospital stays, and higher rates of re-operation [36]. SSIs are the third most costly healthcare-associated infection on an individual basis, with an annual burden of 33.7% of the total annual cost [36]. In another study, 707 individuals from The Health Improvement Network database whose surgical wounds had not healed within 4 weeks post-surgery were analyzed. At discharge, 13% were clinically infected. However, 55% were prescribed antimicrobial dressings or antibiotics. During the next 12 months, 23% subsequently developed an infection, and 19% were re-admitted. The National Health Service average cost for wound care over a period of 12 months was £7300 per wound, with a range of £6000 for healed wounds to £13,700 for unhealed wounds. The mean cost to manage a wound with no evidence of infection was ~£2000, and the conflated cost of managing a wound with an assumed infection was between £5000 to £11,200 [37]. In a systematic review of patients in European hospitals who developed an SSI, independent of the surgical sub-specialty, constituted a financial burden almost twice that of patients without an SSI, and the length of hospitalization was more than two-times longer for patients with an SSI compared to patients without infections [27].

## 5. Use of Fluorescence Imaging to Improve Wound Bacteria Detection

It is costly to wound clinic healthcare systems and payers if wound size and infection are not effectively documented while also creating an economic burden on wound care patients [28,38]. Since delayed wound healing is an indicator of infected wound, visual assessment using wound mapping tools that include wound tracing, scaled photographs, and planimetry monitor wound size changes and have been used to detect wound infection indirectly. These visual assessments can be subjective and imprecise. Ruler-based methods are rapid and readily available but wound area is overestimated by more than 40% and lack consistency in how the wound is measured from week to week, and between clinicians [24]. Hand-written documentation of wound area and status of bacteria tends not be complete, can be easily misplaced, and still requires dictation for electronic medical record (EMR) entry. More accurate, indirect methods are wound area tracings of digital photographs and wound measurement by digital planimetry, which are EMR compatible but time-consuming and involve impractical uploads of clinical data. Wound malodor and wound culture have also been performed when there is suspicion of an infection based on visual observation [3,39]. The Levine swabbing technique may be suboptimal because it primarily collects surface-level bacteria, which may not represent the pathogenic organisms causing infection beneath the wound bed [40]. In contrast, FLI offers a non-invasive, real-time way to detect bacterial presence in vivo, by identifying endogenous bacterial fluorescence, making it more accurate for guiding treatment decisions. Lastly, processing swabs from wounds is laborious and necessitates financial resources [41].

Fluorescence imaging (FLI) is useful for detection of elevated bacterial burden immediately and reliably at the point of care (POC) and can overcome hurdles of accurate documentation. FLI identifies bacterial presence by detecting fluorescence signatures from endogenous porphyrins produced by bacteria, often before clinical signs of infection appear. It provides a noninvasive way to correlate fluorescence and bacterial burden [8,42]. Principles of bacterial auto-fluorescence are exploited by illuminating the wound area with a safe violet light, which reveals endogenous fluorescence signals from tissues and bacteria [43]. The technique documents wound measurement and bacterial status in image format at the patient bedside, reducing risk of errors and eliminating time-consuming additional steps [44]. FLI can also be used to detect bacterial load from biofilms via autofluorescence [45].

## 6. MolecuLight Has Developed Two POC Wound Imaging Devices

**Figure 3:**
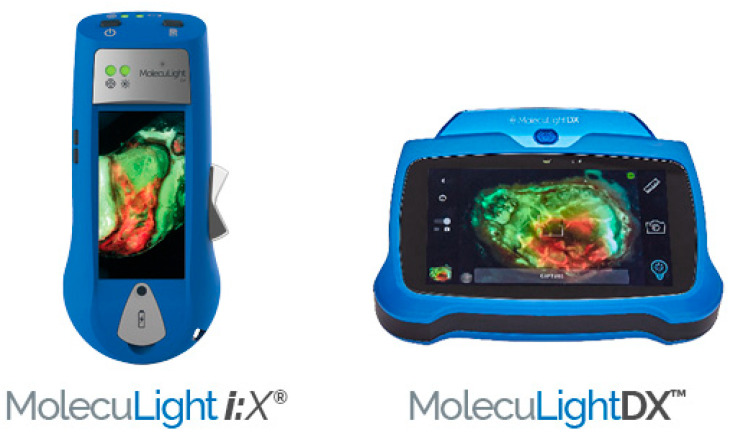
MolecuLight ***i**:X*™ and MolecuLight**DX**™ for detection of elevated burden of wound bacteria to help with clinical decision-making [49].

MolecuLight, a Canadian medical imaging company, offers pioneering POC FLI technology. Both MolecuLight ***i****:X*^®^ and MolecuLight**DX**™ are novel, non-contact, noninvasive, portable devices that capture high-quality images, measure wound areas (length, width, and depth) allowing the real time evaluation of bacterial environment [4,46]. The handheld imaging technology shown in Figure 3 uses safe violet light (405 nm) for excitation of bacterial fluorophores to detect elevated amounts of surface and subsurface (up to 1.5 mm) bacteria in and around the wound bed. Red or cyan fluorescence signals in wounds, detected as emissions at a wavelength of ~510 nm (green/cyan) or 600–700 nm (red), indicate the presence of bacteria above 10^4^ CFU/g. The sensitivity to detect bacterial loads using CSS in combination with FLI is 4 to 11.3 times higher than use of CSS alone [47]. The positive predictive value of CSS + FLI ranges from 93% to 100% [4,43,48]. Red fluorescence is observed in wounds due to the presence of numerous Gram-positive and -negative bacteria, aerobes, and anaerobes. Cyan fluorescence is specific to *Pseudomonas aeruginosa*. FLI can greatly improve wound assessment when used with standard of care (SOC) but should not take the place of CSS with or without microbiological analysis. It should instead serve as an additional tool that the clinician is able to use for diagnosis.

MolecuLight**DX** EMR integration and sticker-less wound measurement capabilities, making it particularly suited for hospital settings, while MolecuLight ***i**:X*™ is considered a more standard device for wound imaging designed for mobile providers or standalone practices. In addition, MolecuLight**DX**™ provides a patient-centric user interface and workflow that permits easy wound tracking for each patient [50].

In 2020, the American Medical Association established two Level III current procedural terminology (CPT) codes to describe noncontact real-time fluorescence wound imaging, for presence, location and load of bacteria per session. CPT 0598T is used for the first anatomic site and CPT 0599T is used for each additional anatomic site [51].

## 7. US Food and Drug Administration (FDA) 510 (k) Clearance for MolecuLight

MolecuLight ***i**:X*™ and MolecuLight**DX**™ are FDA 510 (k)-cleared, first-in-class, patented POC, broad-spectrum FLI medical technologies designed to detect the presence of wound bacteria count greater than or equal to 10^4^ CFU/g, which is defined as the chronic inhibitory bacterial load when there is a chronic presence of bacterial microorganisms in a wound or its surrounding tissue at loads that can damage tissues and be inhibitory to healing, as well as require clinical intervention, with or without presence of clinical symptoms. Studies have pointed to this threshold as being the point at which healing is impaired [8]. Since this was first-in-class technology, there was no predicate device; MolecuLight went through the De Novo classification pathway, and clearance was received in August 2018 [51].

**MolecuLight *i*:X^®^ and MolecuLightDX™ 510(k) Clearances**

○ **191371:** Enables real time POC visualization of fluorescence in wounds, and measures wounds and digitally records all images and area measurements. The MolecuLight ***i**:X*™ fluorescence image, when used in combination with CSS, has been shown to increase the likelihood that clinicians can identify wounds containing bacterial loads of >10^4^ CFU/g as compared to examination of CSS alone [52].
○ **K210882:** Identify areas of wounds containing more bacterial species, including key target pathogens of interest to the CDC that are major causes of antimicrobial resistance. Detectable species include Gram-negative and Gram-positive species, aerobes and anaerobes [53].
○ **K213840:** Identify areas of wounds containing more bacterial species, including key target pathogens of interest to the CDC that are major causes of antimicrobial resistance. Detectable species include Gram-negative and Gram-positive species, aerobes and anaerobes [54].
○ **K211901:** The proposed MolecuLight**DX**™ is considered to be substantially equivalent to the MolecuLight ***i****:X*^®^ predicate device (K191371) [55].

## 8. Brief Summary of Attributes and Benefits of MolecuLight FLI Technology

- Provides a safe, objective, highly sensitive, and easy-to-use portable device to identify clinically significant wound bacterial burden and tissue viability in real-time at the bedside, increasing accessibility and clinical adoption.
- Augments traditional wound evaluation methods by providing objective, visual biomarkers that aid early intervention, guide treatment, and monitor healing progress.
- Delineates wound margins and necrotic areas, assisting in debridement and treatment planning.
- Facilitates early detection and removal of bacterial load to reduce wound infection and allow for faster wound healing.
- Allows better treatment monitoring by imaging changes in fluorescence over time; clinicians can assess response to interventions such as antibiotics or dressings.
- Measures wound area (length and width), allowing clinicians to digitally measure a wound, save the measurement, save the bacteria location, and allow access to images longitudinally to assess wound healing.
- Supports documentation for wound monitoring and reimbursement as part of an individualized treatment plan.
- Utilizes information to manage bacterial burden and more effectively use skin substitutes/CTPs and other adjunctive treatment modalities.

## 9. Overview of Procedure for Bedside FLI

**Figure 4:**
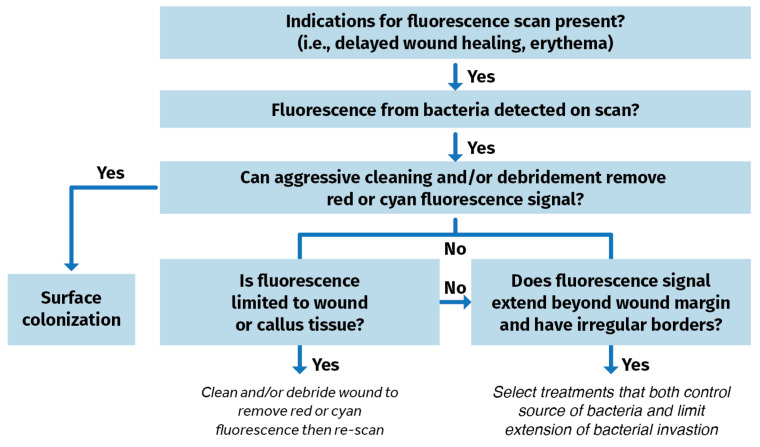
Clinical decision tree using fluorescence imaging information to identify bacterial burden in wounds [56].

Blood or debris are wiped away from the wound(s). The patient is then positioned for imaging. The area is made dark. A dark drape is positioned over the wound and device to create a dark environment if turning off room lights is inadequate to sufficiently visualize FLI signals. The device is situated parallel to, and at an appropriate distance from the site of the wound. The violet light on the device is turned on and the clinician focuses on the wound before capturing the fluorescence image. POC signals are captured, delivering and documenting immediate information on the bioburden. A clinical decision-making tree is shown in Figure 4.

## 10. Total Time for MolecuLight Procedure

The time required to perform patient positioning, device setup, FLI of wound bed, interpretation, diagnosis, and cleanup is on average 28 min per patient encounter for a single wound in an otherwise healthy patient [57]. Several factors can affect the time of each encounter (Table 2).

## 11. Delphi Panel Recommendations for Appropriate Use and Impact of FLI

The Delphi Panel (N = 32) wound experts (56% MDs, 22% podiatrists, 12.5% nurses/nurse practitioners) representing many sites of service. The panel has considerable clinical experience with FLI [58]:

- 96% of experts from the Delphi Panel indicated that use of imaging-informed treatment plans led to improved wound healing.
- >80% reported treatment plan changes.
- 78% indicated imaging reduced their rates of amputation.
- 83% reported reduced rates of microbiological sampling.

The panel also provided recommendations on the appropriate use and frequency of FLI. The panel states that the FLI procedure should be done at baseline, and repeated during the first 4 weeks, and/or if one observes an increase in wound size. If a wound meets the criteria for imaging (e.g., CSS) or if a wound exhibits positive fluorescence, then FLI should be performed weekly. If symptoms develop or change, that is an indication of the need for FLI [58].

## 12. Competencies Needed to Perform FLI of Bacterial Burden

Fundamental competencies to perform FLI include the ability to setup the device (focus image, download software, export images), capture images, understand use of range finder and the light indicators, know how to appropriately adjust position and distance between the patient and the imaging device, establish appropriate lighting conditions, and interpret images showing red or cyan fluorescence. Advanced competencies include ability to interpret images to plan the patient’s treatment and align image interpretation to location of elevated levels of wound bacteria [58].

These competencies are achieved through training on the MolecuLight imaging devices, provided by the manufacturer. There are three stages of the training: online self-paced eLearning, including certification test (~2.5 to 3.0 h); live virtual training (1.0 to 1.5 h), and onsite training done over the course of at least one clinic day with patients. Image interpretation resources, user instructions, and training videos are provided. However, interpretation is prone to subjectivity due to multiple human and technical factors. Sources of subjectivity include visual analysis bias of fluorescence intensity and distribution; color perception differences, especially under varying lighting conditions and skin tones; and observer expectations. Technical factors include spectral overlap when several bacterial species produce fluorophores in overlapping wavelengths and autofluorescence from tissue or dressings. Other instrument limitations may include variability in how the camera is positioned relative to the wound and non-uniform excitation light or poor camera focus.

Panelists defined clinical indications for use of FLI to detect wound bacterial burden. Experts agreed that review of a patient’s medical history could point to certain comorbidities, e.g., diabetes that can mask CSS of infection. Delphi panelists unanimously agreed that DFUs should be imaged. Medical history can also reveal past problems with delayed wound healing, failure of prior wound treatments, positive CSS, or autoimmune disorders that all point to a need for FLI.

All but one panelist agreed that clinical assessment and observation of the presence of just one CSS of infection (e.g., local warmth, new or increased localized pain, erythema, purulent discharge, increased malodor, extended thickened and hardened) indicates a need for FLI.

There was also high consensus that certain procedures and treatments, such as debridement, use of antimicrobials, CTP or graft application, warrant FLI. Lastly, wound sampling and positive microbiology is considered a reason to perform FLI [58].

Since FLI uses safe, visible light for bacterial detection, imaging can be done at baseline and repeatedly, as needed, depending on clinical status. The recommended frequency is weekly if the wound at baseline is positive for fluorescence, or if patients show CSS, or repeated during the first 4 weeks if there is an increase in wound size. Timing of repeat FLI requires some level of clinical judgment [58].

Support for FLI can be found in several other guidelines, as follows:

1. **International Surgical Wound Complications Advisory Panel (ISWCAP).**

ISWCAP issued a consensus document in 2022, “Optimizing Prevention of Surgical Wound Complications: Detection, Surveillance, and Prediction.” The ISWCAP expert group agreed that POC FLI is a diagnostic technology that could provide significant benefit in the early identification of SSI and other surgical wound complications. They noted that several studies have established the role of this technology in the detection of chronic wounds. SSI detection by FLI is an emerging field with promising initial results including an 11-fold greater sensitivity in the detection of infection compared to CSS alone [59,60].

ISWCAP also issued an additional global guideline for postoperative incision surveillance and care noting that the use of FLI to detect bacterial load and track its location can result in improved interventions, informing appropriate wound cleansing and debriding techniques, as well as the use of topical antimicrobial therapies. It can also help reduce the overuse of systemic antibiotics, which may lower antibiotic resistance [61].

2. **International Wound Infection Institute (IWII).**

IWII Consensus Update 2022 states that POC tools for detection of wound infection are becoming more readily available and accessible. The handheld FLI devices provide information on bacterial burden in wounds in real time through detection of bacterial fluorescence. Recent studies have reported that the device has a PPV of >95% for detecting presence of moderate to heavy bacteria load within the wound area [12].

3. **JWC International Consensus Document.**

The Wound Repair and Regeneration Guidelines state that FLI, a relatively new technology might help with the pre- and post-intervention wound assessment, can provide information on the presence, location, and type of bacteria in a wound—information that is useful towards predicting potential to heal, identify factors impeding wound healing, and guide management plans [62].

A second consensus statement appeared in the Leg Ulceration in Venous and Arteriovenous Insufficiency Guidelines stating that, if available, FLI can help determine the location of bacterial activity, the species, the amount of bacteria and the presence of biofilm, thereby facilitating earlier and more thorough debridement of biofilm and non-viable tissue. The non-invasive nature of FLI allows this diagnostic technology to be helpful for those diagnoses where the alternative of performing a biopsy could be detrimental, such as in pyoderma gangrenosum, as this can trigger an exaggerated inflammatory response and worsening of the wound area [63,64,65].

## 13. Summary of Clinical Evidence to Support FLI

Prospective and retrospective clinical studies published in peer-reviewed journals are recorded by site of wound (s) and summarized in Table 3, providing evidence for the value of FLI of bacteria in wounds. Although many of the studies listed come from well-controlled, multi-site, clinical trial settings, some studies have small sample sizes, are single-center studies, or focus on specific wound types. Fluorescence wound imaging using MolecuLight technologies has resulted in physicians changing treatment plans and more efficiently using advanced wound technologies such as autologous grafts, CTPs/skin substitutes, NPWT, and hyperbaric oxygen therapy, among others. Real-world studies using FLI technology report promising improvements in patient outcomes, less waste of resources, and reduced total cost of care [7,34,66,67]. Studies over the past 8 years have demonstrated that FLI provides multiple benefits for many wound types [68].

**POC detection at the bedside is increased.** FLI demonstrated increased sensitivity, specificity, and accuracy for a wide variety of wounds, including perineal and SSI [60,69,70]. Okeahialam et al. reported sensitivity of 83% and specificity of 90% in patients with perineal wound infections [71]. In another study, published by Farhan and Jeffery, sensitivity (89%), specificity (87%), accuracy (86%), positive predictive value (87%), and negative predictive value (78%) for detection of wound bacterial infection with FLI are high [27]. In a study comparing FLI with CSS reported by Le et al., the use of the FLI diagnostic procedure to detect bacterial loads resulted in 4-fold higher sensitivity and 2.2-fold higher accuracy in wounds that would have been missed if assessed by CSS alone [4]. Furthermore, sensitivity and accuracy were improved when FLI was combined with CSS compared with CSS alone for identifying wounds with moderate-to-heavy loads of bacteria (*p* = 0.002) [3], and infection detection sensitivity in pediatric burns improved by 39% [72].

Bacterial load and biofilm can be detected even in patients who are asymptomatic, when detection with SOC methods have failed, thereby complementing and improving on detection based on visual signs and symptoms. FLI provides objective information to enhance routine wound assessment by identifying bacterial loads missed by CSS alone in 47% of wounds [73] and bacterial biofilm missed by standard clinical assessment or biofilm blotting [45] and alter treatment plans in 56% to 73% of wounds [74].

**Ability to modify the wound treatment plan.** Compared with clinical judgment alone, the more accurate and relevant microbiological profile guides optimal wound swab sampling for microbiological analysis [25] and can guide curettage or biopsy and potentially reduce false-negative sampling [10]. It also allowed for rapid initiation and more aggressive cleaning and debridement [46,56,64,65], as needed, and appropriate selection of antimicrobials and other treatments [7,34], or the selection of specialized dressing [46] and the frequency that NWPT dressing change was needed [44]. The ability to assess efficacy of debridement of wound bed and peri-wound bacterial burden improved with FLI [69]. FLI is also useful for wound preparation before application of CTP for wounds such as DFUs [14] and for predicting outcome of skin grafts among burn victims and decisions regarding grafts to improve outcomes [15].

**Management changes resulting from FLI lead to improved clinical outcomes.** Elimination of bacterial fluorescence leads to greater wound area reduction indicative of a healing trajectory [23,24,66]. In a randomized controlled trial, addition of FLI to SOC for DFUs allowed for debridement in wounds with positive fluorescence, thereby accelerating wound healing and reducing wound area [66]. Incorporating FLI into routine practice for DFUs was associated with a reported 27% increase in the number of wounds with bacterial burden that were detected, resulting in a 33% decrease in expenditure for antimicrobial dressing. This reduction included a 49% decrease in the percentage of patients prescribed antimicrobial dressings and a 33% decrease in antibiotic prescriptions for wound management, which led to a 23% increase in wounds healed within 12 weeks (48% vs. 39%) [7]. A retrospective interventional study to assess the impact of FLI on personalized pressure wound treatment plans and outcomes reported 71.0% more wounds healed by 12 weeks in the FLI cohort (38.5% with FLI vs. 22.5% in the SOC cohort). Wounds in the FLI cohort healed 27.7% faster (−4.8 week), on average, and they were 1.4 times more likely to heal [68]. In addition to faster healing rate, these patients exhibited improved infection control, reduced reinfection rate [61], reduced duration of antibiotic use and costs [75], and reduced infection-associated complications such as cellulitis, osteomyelitis, or wound-associated hospitalization [68,75].

**Figure 5:**
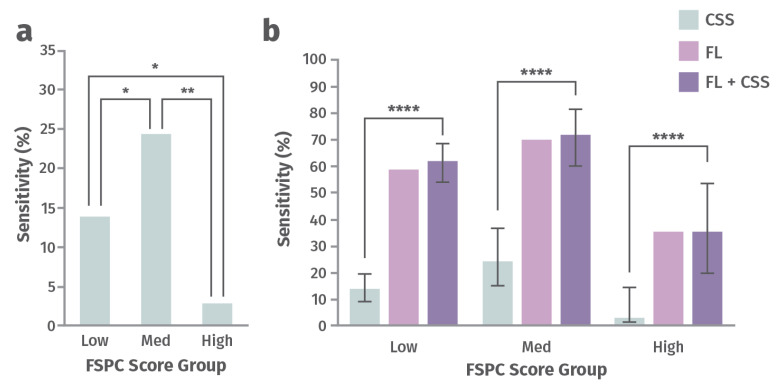
Sensitivity for detecting a high load of bacteria with (**a**) clinical signs and symptoms (CSS) alone and (**b**) CSS alone vs. fluorescence imaging (FLI) alone vs. CSS in combination with FLI for groups of patients with low, medium, and high Fitzpatrick Skin Phototype Classification (FSPC) scores [76]. Statistically significant at *p* < 0.05 (*), *p* < 0.01 (**), or *p* < 0.0001 (****).

**Addresses racial disparities in wound management.** A post hoc analysis of 350 chronic wounds from a prospective 14-site clinical trial aimed to determine how perception of CSS of infection differs as a function of a patient’s skin tone and whether FLI can offer a more objective diagnostic solution [76]. Participants were assigned to one of three groups, depending on the amount of their skin melanin, as measured by the Fitzpatrick Skin Phototype Classification (FSPC) system: low (Type I and II), medium (Type III and IV), high (Type V and VI) [76]. CSS and total bacterial load (TBL) were compared across these groups (Figure 5). Sensitivity for detection of TBL >10^4^ CFU/g by assessing CSS alone or CSS in combination with FLI of wound bacteria were compared in this study. The ability to predict burden of bacteria using CSS (including erythema) was significantly lower for patients with higher melanin content and increased by up to 12-fold after incorporating FLI [76]). Missed diagnosis of bacteria and infection could delay initiation of treatment, increase the risk of complications, and increase the risk of worse wound outcomes. FLI helps fill this gap and serves as a more objective and equitable indicator of presence of wound bacteria [76].

These findings underscore the problem of health inequities in wound care as a function of differences in skin tone, where missed diagnosis of wound bacteria and infection could delay treatment, and increase the risk of complications and poor outcomes.

## 14. Economic Value of Real-Time Wound FLI Technology

In addition to wound management clinical benefits from FLI to detect clinically relevant bacteria and biofilm in wounds leading to actionable management changes, it also reduces healthcare spending (Table 4). Reductions in costs include those for laboratory tests for confirmation of microbial presence of a wound [67], antibiotic prescriptions [7,75,77], as well as wound care procedures, antimicrobial dressing expenditure [7], and time and supplies for NPWT dressing changes [44]. The costs to manage infection-associated complications (cellulitis, osteomyelitis, gangrene) and need for intravenous antibiotics and wound-associated hospitalization and sepsis also decline [56,68]. Another cost savings from FLI is the reduction in the number of failed CTPs/reduced cost of CTPs, which can now be used only when the wound bed is ready [78].

## 15. Limitations of FLI for Detection of Wound Bacterial Infections

Although FLI provides significant clinical and economic benefits in wound care, there are limitations to its use, and it should therefore not take the place of CSS with microbiological analysis but instead serve as an additional tool for diagnosis. Limitations to FLI include the following [22,79,80,81]:

- FLI requires appropriate darkness to capture optimal fluorescence images. Ambient light contamination can lead to inappropriate interpretation of images. Use of a MolecuLight DarkDrape^®^ attachment can provide the required darkness to perform FLI properly.
- Accurate fluorescence image interpretation has a learning curve. New users of the technology may find it challenging to differentiate the cyan fluorescence from *P. aeruginosa* from the green fluorescence from endogenous structures. Continued use and experience with the device and utilization of image interpretation resources can help.
- Accurate assessment requires good imaging practice such as wound cleaning, removing as much blood as possible, and removing imaging artifacts from white bedsheets and gauze bandages. Blood can absorb the violet excitation light and mask other fluorescence signatures.
- Color-blind individuals cannot interpret fluorescence images accurately as a result of the high proportion of red and green colors.
- Violet excitation light cannot penetrate >1.5 mm into the skin. Some subsurface bacteria can be detected, but the presence of bacteria located deeper within the wound tissue may not be visible, including infections that are deep tunneling.
- Fluorescence signals associated with bacteria do not provide an exact numerical estimate for the bacterial load in a wound other than indicating it is above the chronic inhibitory bacterial load, which is considered the tipping point between requiring vigilance of the wound to requiring intervention to address the bacterial load.
- Aside from *Pseudomonas*, fluorescence signals associated with bacteria cannot determine the specific bacterial species within the wound or the antibiotic susceptibility of these microorganisms. Toward that end, swab or tissue sample microbiological analysis is needed.
- Most chronic wound infections are polymicrobial.
- A small subset of bacteria are not detectable with FLI. These include *Streptococcus* and *Enterococcus* species.
- To guarantee optimal recovery from all bacteria, collected swab samples should be transferred for microbiological analysis, typically within 4 h.

## 16. Other FLI Technologies Under Development

A company called Designs for Vision Inc. has introduced a new device called REVEAL, which is an autofluorescence imaging form factor. This device can be worn on top of a pair of glasses, which the physician could wear during surgery. The device does not require specific lighting conditions, and a handheld camera is not needed to interpret the results, thus allowing use of the device during the process of active surgical debridement [81].

## 17. Summary

Undetected bacterial loads in acute and chronic wounds lead to healing delays and missed opportunities for appropriate treatment. The ability to detect asymptomatic bacterial load and biofilm incidence using FLI technology provides multiple benefits that extend to patients, providers, and payers. Detection of wound bacterial burden with FLI has important diagnostic and treatment outcomes with the potential to improve overall well-being of the patient, reduce healthcare disparities, and decrease cost of care.

- Improves diagnostic procedures examining and managing complex wounds at the POC [3,46].
- Results in positive prediction of the presence of bacteria at potentially harmful levels, thus reducing the risk of errors in sampling with image-guided curettage or biopsy [43].
- Four-fold improvement in detecting chronic inhibitory bacterial loads of >10^4^ CFU/g over CSS alone that impair wound healing and lead to CTP/graft failure [4,8,15].
- Reduces racial and ethnic disparities due to variations in skin pigmentation; significant improvement in detection of high bacterial burden independent of skin pigmentation [56,67].
- Improves ability to monitor surgical sites, which may reduce the rate of surgical site infections and its sequelae [60].
- Results in informed diagnoses and frequent, r.zeal-time treatment plan changes post-imaging that better align with wound needs [46].
- Can guide extent of wound bed cleaning and debridement, need for and timing of dressing changes, and the selection of appropriate and cost-effective NPWT [4,64,65,82].
- Leads to proactive, targeted management of wound-related cellulitis and reduction in antimicrobial overuse [4,56].
- Reduces the need for antimicrobials and antibiotics leading to antimicrobial stewardship [34,73].
- Reduces wastage and overuse of skin substitute grafts and other expensive advanced therapies [15,78].
- Guides the placement of skin substitutes for optimal graft take, thus reducing wastage and overuse of expensive wound treatment technologies [14].

## Figures and Tables

**Table 1:** Methods of Detection of Bacterial Burden in Wounds [3].

	Method	Strengths	Limitations
Visual assessment	Clinical signs and symptoms	Simple	Subjective poor sensitivity, especially in chronic wounds
Microbiological analysis	Swab culture (e.g, Levine)	Non-invasive, simple, inexpensive	Often misses deeper pathogens Results take days
	Tissue biopsy	Gold standard for bacterial quantification	Invasive Painful Risk of bleeding Culture-dependent
	Quantitative culture		Cannot detect unculturable organisms
Histopathology	Histology Immunohistochemistry	Assesses tissue morphology and bacterial presence	Requires excised tissue Lab processing
Assay to detect molecular or biochemical markers	Bacterial DNA, MMPs, inflammatory cytokines	Rapid, highly sensitive	Cannot differentiate live/dead bacteria Expensive
Advanced diagnostic imaging	Fluorescent imaging	Real-time detection at POC Detects clinically relevant thresholds Detection below signs of infection	Requires training May miss anaerobes or biofilm Some false positives and negative due to tissue or dressing mimicking bacterial signals
Biosensors	Detect pH, temperature, oxygen	pH: Early detection, passive mechanism Temp: Easy to interpret, cost-effective	Early-stage development, limited validation Non-specific (can be altered by other factors) May be prone to false positives
Smart dressings	Microbial enzymes or toxins	High sensitivity	Needs electronics integration
Biofilm Detection		Enables targeted debridement and topical therapies early, reducing chronicity and promoting healing	Technically demanding, mostly research-based, No universally accepted bioburden threshold, Sampling can result in inconsistent detection May not recover dormant bacteria

**Table 2:** Factors Resulting in Variability of Encounter Time With Each Patient.

Time to address a small single wound, large wound, or multiple wounds located close together or multiple wounds at a distance from each other. Time to locate wound (s), assess type of wound, address patient mobility and flexibility, cognitive ability, and limitations of higher body mass index. Number of repeat images or videos required, i.e., if more images than before and after wound bed preparation are required or wound cannot be captured by a single image. Time to diagnose presence of bacteria (location, quantity, species). Time to interpret images and plan treatment. Time to upload all images/videos/data to the electronic medical record.

**Table 3:** Prospective and Retrospective Studies Supporting Point-of-Care FLI to Detect Wound Bacterial Burden.

Country	Study Type/Design	Findings		
**Mixed Chronic Wounds**				
**USA**	Prospective real-world study (N = 1447) Trafelet, N., et al., 2024 [29]	Addition of FLI in outpatient wound care centers provided actionable information at the bedside in 8 outpatient wound clinics, leading to a significant decrease in the use of systemic antibiotics (8% vs. 36% for standard of care [SOC] alone) and a decrease in other antimicrobial prescriptions. Use of oral and topical antibiotics were also reduced in the SOC + FLI cohort. FLI allows for integration of antibiotic stewardship into healthcare system.		
**USA**	Post-hoc analysis from a prospective cohort trial (N = 350) Johnson, J., et al., 2024 [58]	Evaluated diagnostic accuracy of MolecuLight imaging across skin tones. FLI improved the detection of high bacterial load in each group vs SOC across all skin tones; most pronounced in high skin pigmentation group with a 12-fold increased sensitivity over standard infection assessment alone. Erythema reported less often with increasing Fitzpatrick Skin Prototype Classification (FSPC) score (*p* = 0.05): from 13.4% (low), to 7.2% (medium), to 2.3% (high), despite similar bacterial loads (median = 1.8 × 10^6^ CFU/g). Clinical signs and symptoms (CSS) sensitivity in high group (2.9%) was 4.8-fold to 8.4-fold lower than the low (*p* = 0.003) and medium groups (*p* = 0.04).		
**USA**	Multi-center prospective single-blind cohort trial (N = 350) Le, L., et al., 2021 [1]	MolecuLight FLI increased point-of-care (POC) detection of wounds with high bacterial loads (>10^4^ CFU/g) by 4-fold vs SOC. Downstream patient care affected: sampling location (44.6% of wounds), cleaning (42.9%) and debridement (48%), selection of antimicrobials (53.1%) and other treatments (55.4%). 82% of study wounds (287/350) had clinically significant bacterial loads (>10^4^ CFU/g) that were missed by SOC.		
**Germany**	Prospective clinical study (N = 151) Moelleken, M., et al., 2024 [59]	FLI before and after SOC mechanical debridement showed a significant reduction of bacterial colonized areas by an average of 29.6% in the wounds, 18.9% in the wound edges, and 11.8% in the wound surroundings.		
**Ireland**	Prospective 2-week study (N = 27) Derwin, R., et al., 2023 [19]	Clear relationship exists between FL signals and wound area reduction (WAR), suggesting that wound assessment, time for wound bed preparation, and treatment planning can be further enhanced by technology such as FLI. Overall average WAR was: −3.80 cm^2^, or a decrease of 46.88% (manual measurement) −2.62 cm^2^, or a 46.05% decrease (digital measurement with MolecuLight) Wounds exhibiting initial bacterial loads that resolved by the final visit exhibited the most favorable WAR, and wounds with persistent bacterial loads from the outset to the final visit demonstrated less favorable WAR.		
**USA**	Quantitative comparative study (N = 71) Oropallo, A., et al., 2024 [60]	FLI demonstrated that vigorous mechanical gauze cleansing outperforms wound soaking in removing bacterial loads at or just beneath the skin surface. FLI showed that there were no significant differences in effectiveness of 7 cleaning solutions; antiseptic cleaners removed more bacterial loads than non-antiseptics.		
**Ireland**	Prospective single blind observational study (N = 33) Hurley, C.M., et al., 2019 [42]	FLI has high sensitivity (100%), specificity (78%), positive predictive value (PPV; 95.4%), and negative predictive value (NPV; 100%) for real-time detection of harmful wound bacteria, aiding in precise clinical decision-making regarding use of antibiotics and specialized dressings.		
**Canada**	Prospective validation trial (N = 28) Raizman, R., et al., 2021 [43]	POC FLI alongside CSS can enhance detection of *Pseudomonas aeruginosa* (PA) in wounds with a high PPV (92.9%) and can provide immediate information on efficacy of treatments targeted to eradicate PA. Bacterial load from cyan-positive regions ranged from light to heavy. <20% that were PA-positive exhibited the classic symptoms of PA.		
**USA**	Prospective observational study (N = 11) Cole, W., Coe, S. 2020 [18]	FLI as part of routine wound care enables proactive management. Bacterial fluorescence was present in 10 wounds and persisted for an average of 3.7 weeks. The presence of bacterial red or cyan fluorescence (FL) correlated with an average increase in wound area of 6.5% per week, indicating stalled or delayed wound healing. FLI improved treatment decision-making that may help accelerate healing of hard-to-heal wounds, assisting in determining location and extent of wound debridement, and selecting dressings and/or antimicrobials. Elimination of bacterial FL with targeted debridement and other treatments correlated with an average WAR of 27.7% per week (*p* < 0.05), indicative of a healing trajectory.		
**USA**	Retrospective single time-point, large scale observational study (N = 1000) Jacob, A., et al., 2023 [40]	Data, from a range of wound types, facilities, and clinician skill sets, demonstrated that POC FLI improves infection management. FLI signals indicating elevated bacterial loads present in 701 wounds (70.8%), while only 293 (29.6%) showed CSS of infection. Upon visualization of elevated bacterial loads in wounds via FLI, clinicians amended at least one aspect of their treatment plan for 528 wounds (53.3%) as follows: – FL-targeted debridement (17.2%) – More extensive debridement (18.7%) – More extensive hygiene (17.2%) – New topical therapies (10.1%) – New systemic antibiotic prescriptions (9.0%) – FL-guided sampling for microbiological analysis (6.2%) – Changes in dressing selection (3.2%)		
**Taiwan**	Single center retrospective analysis (N = 33) Li, T-H., et al., 2025 [61]	Use of FLI improves outcomes in deep sternum wound infections. White blood cell (WBC) counts, C-reactive protein (CRP) levels, duration antibiotic use, antibiotic costs, reinfection rate, and osteomyelitis recurrence rate improved in real-time FLI group (<0.001, <0.001, 0.042, 0.022, 0.049, and 0.022, respectively). Length of intensive care unit stay and duration to complete healing decreased (<0.001 and 0.046). – Infection control group was 25.67 times more likely to have their WBC count return to 4000–10,000/μl within 7 days (9.1% vs 72.7%; *p* < 0.01; OR = 26.67) and ~7 times more likely to have CRP levels return to <1 mg/dl within 14 days (31.8% vs 78.8%; *p* < 0.01; OR = 7.959) than the hyperbaric oxygen therapy (HBOT) group. – Average duration antibiotic use: (29.5 vs 22 days; *p* = 0.042) and antibiotic cost ($1643.78 vs $573.57; *p* = 0.022) were lower in FLI group than in HBOT group.		
**UK**	Literature review Farhan, N. Jeffery, S. 2021 [22]	Meta-analysis of 11 studies on the diagnostic accuracy of FLI for detecting wound bacteria in burns showed: – Sensitivity 89%; Specificity 87%; Accuracy 86% – PPV 87%, NPV 78%		
**USA**	Literature review Caputo, W.K., et al., 2022 [28]	Antibiotic prescribing rates were very high (53.3% to 71.0%) among outpatient wound care patients By using an FLI-informed approach: – Hygiene-based decisions (debridement, cleansing) were impacted in 70% of wound visits, – Antimicrobial prescribing decisions changed at the bedside in 41% of visits, and – Systemic antibiotic use was greatly reduced.		
**Diabetic Foot Ulcers (DFUs)**				
**UK**	12-week, prospective, double-blinded randomized controlled trial (N = 56) Rahma, S., et al., 2022 [62]	DFU healing rate in those with positive FLI and those with SOC assessment showed: Healing favored those with FLI vs SOC. 2-fold increase in DFU healing rate at 12 weeks in FLI group: – 45% in the auto-fluorescence arm – 22% (n = 6 of 27) in the control arm Better wound area reduction in FLI group: – 40.4% (autofluorescence) vs 38.6% (control) at 4 weeks – 91.3% (autofluorescence) vs 72.8% (control) at 12 weeks (25% improvement) The antibiotic prescription rate was kept under 10% in the FLI group.		
**USA**	Post-hoc analysis of prospective cohort trial (N = 138) Armstrong, D.G., et al., 2023 [4]	Chronic inhibitory bacterial load (CIBL) describes frequently asymptomatic, high bacterial loads in DFUs and peri wound tissues, which require clinical intervention to prevent sequelae of infection. – FLI can be used to detect CIBL at the bedside. Bacterial presence confirmed in 131/138 DFUs. Of these, 93.9% had loads >10^4^ CFU/g. – In those wounds, symptoms of infection were largely absent and did not correlate with, or increase proportionately with, bacterial loads at any threshold (10^4^ up to >10^8^ CFU/g). FLI increased sensitivity for the detection of bacteria across loads 10^4^–10^9^ (*p* < 0.0001), peaking at 92.6% for >10^8^ CFU/g.		
**USA**	Prospective pilot study (N = 11) Ai-Jalodi, O., et al., 2021 [9]	FLI is highly predictive of DFU healing outcomes with porcine-derived skin substitutes (cellular and tissue-based products [CTP]/skin substitute and autologous skin graft). – DFUs that healed were positive for bacterial FL on Day 0. – In all cases FL disappeared during the trial, attributed to management interventions. – If bacterial FL persisted throughout the trial, then those DFUs did not heal.		
**UK**	Retrospective pre/post intervention cohort outcomes study (N = 229) Price, N. 2020 [63]	FLI is useful when examining and managing complex foot wounds at the POC, helping to make decisions and supporting discussions within the wider multidisciplinary team. Implementation of FLI resulted in a: – 33% decrease in annual antimicrobial dressing expenditure – 49% decrease in prescription of antimicrobial dressings – 33% decrease in antibiotic prescriptions 48% positive for FL vs 39% with SOC were healed at 12 weeks (23% improvement) likely due to earlier bacterial detection and improved wound hygiene.		
**Canada**	Non-randomized clinical trial (N = 33) Ottolino-Perry, K., et al., 2017 [64]	Compared with Levine technique, real-time FLI of adults with >1 non-healing DFU more accuratelyidentified presence of moderate and/or heavy bacterial load (accuracy 78% vs 52%; *p* = 0.048) and maximized effectiveness of bacterial load sampling with no significant impact on clinical workflow.		
			**CSS**	**FLI**
		Sensitivity	73%	78%
		Specificity	38%	78%
		PPV	44%	64%
		NPV	67%	88%
**Pressure Wounds**				
**USA**	Retrospective pre/post-interventional cohort study (N = 167) Kelso, M., Jaros, M. 2024 [65]	FLI-guided management of pressure ulcers significantly improved healing and infection outcomes in highly complex and multimorbid patients in long-term care or skilled nursing facilities. FLI results support proactive monitoring and management of planktonic and biofilm encased bacteria in a real-world setting. FL-imaged wounds healed 27.7% faster (−4.8 weeks) on average (*p* = 0.043); average duration of healed wounds at any point during the study was 17.2 ± 12.4 weeks for SOC and 12.4 ± 8.9 weeks for FLI cohorts. FLI was associated with 71.0% increase in 12-week wound healing rate (38.5% in FL cohort vs 22.5% in SOC cohort (*p* = 0.007). Infection-associated complications 75.3% lower in FLI cohort (*p* = 0.007); fewer wounds developed cellulitis, osteomyelitis, gangrene, wound-associated hospitalization, and sepsis.		
**Lower Leg Extremity Wounds**				
**USA**	Prospective observational case studies of patients with cellulitis (N = 15 of 236) Andersen, A., et al., 2022 [50]	Patients in an outpatient wound care center identified with cellulitis (15 of 236 patients or 6.4%) using an algorithm including FLI, which resulted in rapid initiation of treatments that resolved the fluorescence. None had worsening cellulitis that might require intravenous antibiotics and/or hospitalization.		
**Canada**	Single-blind clinical validation trial (N = 60) Rennie, M.Y., et al., 2017 [38]	When used with best clinical practices, FLI enabled real-time localization of bacterial presence at clinically significant loads (≥10^4^ CFU/g) with high confidence in lower limb chronic wounds. Image-guided curettage or biopsy positively detected bacteria in chronic wounds at potentially harmful levels, eliminating risk of false negative sampling and enabling informed POC treatment decisions. Scrapings from red FL regions yielded PPV = 100% with mostly moderate or heavy bacterial growth.		
**Germany**	Prospective clinical study (N = 25) Moelleken, M., et al., 2020 [66]	FLI localized bacterial colonized areas and showed persistent peri-wound bacteria post-debridement in venous leg ulcers. Pre-debridement, bacterial FL comprised 10.4% of wound beds and ~25% of the peri-wound area. 99.4% reduction in bacteria in the wound bed and 64.3% in the peri-wound after single mechanical debridement (*p* < 0.001). Across bed and peri-wound, a single SOC mechanical debridement left behind 29% of bacterial FL-positive tissue regions.		
**Perineal Wounds**				
**UK**	Post-hoc analysis of prospective observational study (N = 55) Okeahialam, N.A., et al., 2022 [67]	Bacterial FL detected by MolecuLight was a significant, independent risk factor associated with delayed wound healing in dehisced perineal tear wounds (OR 0.21 [0.05–0.87]). – 30.9% wounds had significant bacterial colonization, identified with FLI. – 47.1% of wounds with significant bacteria colonization healed within 4 wk, vs 78.9% of wounds not colonized (*p* = 0.03).		
**UK**	Prospective validation study (N = 80) Okeahialam, N.A., et al., 2023 [68]	FLI of perineal wounds demonstrated high sensitivity (83%) and specificity (90%) in identifying significant bacterial colonization compared to clinical signs alone.		
**Burn Wounds**				
**Mexico**	Prospective outcomes analysis (N = 38) Hanson-Viana, E., et al., 2024 [10]	FLI is an accurate method for assessing recipient sites and predicting outcomes of skin grafts among burn patients and can inform better decision-making surrounding grafts that may lead to better outcomes. For predicting any type or range of graft loss in the entire cohort, FLI was found to have a: – Sensitivity of 86%; specificity of 98%; accuracy of 94% – PPV of 72%; NPV of 99%		
**Canada**	Retrospective review (N = 178) Turner, E., et al., 2024 [69]	Incorporation of FLI in standard pediatric burn wound assessments can improve the detection of infections, which may promote improved wound healing outcomes and antimicrobial stewardship. FL corresponded with CSS in 81% of wounds and microbial findings in 82% of wounds. FLI in combination with CSS, improved sensitivity for detecting wound infections by 39%.		
**Surgical Site Wounds**				
**USA**	Post-hoc analysis of prospective clinical trial (N = 58) Sandy-Hodgetts, K., et al., 2022 [54]	Advanced imaging of pathological bacterial burden improves surgical site monitoring and may reduce the rate of surgical site infections (SSIs). 75.8% of surgical site wounds had bacterial loads >10^4^ CFU/g (median = 3.11 × 10^5^ CFU/g). Only 3 of 44 were positive for CSS of infection (CSS sensitivity of 6.8%). – FLI improved sensitivity of bacterial detection by 5.7-fold vs CSS alone (*p* = 0.0005). – Sensitivity improved by 11.3-fold over CSS among clinicians highly experienced with FL interpretation (*p* < 0.0001).		

**Table 4:** Economic Benefits of Point-of-Care FLI to Detect Wound Bacterial Infection.

Study	Country	Study Type/Design	Findings
**Routine fluorescence imaging to detect wound bacteria reduces antibiotic use and antimicrobial dressing expenditure while improving healing rates: retrospective analysis of 229 foot ulcers** **Price, N. 2020 [63]**	**UK**	Retrospective pre/post-implementation cohort study of patients with diabetic foot ulcers	Implementation of FLI was associated with a: – 33% decrease in annual antimicrobial dressing expenditure, – 49% decrease in prescription of antimicrobial dressings, – 33% decrease in antibiotic prescriptions, and – 23% increase in wound healing rates within 12 weeks (48% vs. 39%) likely due to earlier bacterial detection and improved wound hygiene. Increased healing rate projected to decrease annual wound costs by 10%.
**Utility of MolecuLight i:X for managing bacterial burden in pediatric burns (N = 16)** **Farhan, N. Jeffery, S., 2020 [75]**	**UK**	Observational study	Real-time confirmation of bacterial status could reduce the need for swabs and would prevent false-negative swabs by targeting a region of the wound with bacterial fluorescence. Potential cost savings in the UK include: – Swab test currently costs around £90, extra £5 for any additionally requested sensitivity test per microorganism. – Evidenced-based dressing selection instead of primarily using silver-based dressings, some of which were unnecessary (cost of >£25 million in 2010).
**Use of a fluorescence imaging device to detect elevated bacterial loads, enhance antimicrobial stewardship, and increase communication across inpatient complex wound care teams** **DasGupta, T., et al. 2022 [76]**	**Canada**	Prospective implementation study at two hospital inpatient sites	FLI supports more judicious prescribing and decreases the use of antimicrobial agents or antibiotics when warranted. Imaging resulted in cost savings of 33% on antimicrobial agents for the year; a 10% total savings per patient also was projected.
Can fluorescence imaging predict the success of CTPs for wound closure and save costs? Aung, B. 2019 [77]	**USA**		Based on two patient case studies, if applied weekly over the course of 4 weeks, the cost of using CTPs is estimated to be over $6400, with an additional $1260 in facility fees and clinician time to manage chronic wounds in an outpatient facility. Being able to predict wound readiness would contribute to cost savings. FLI provides objective information on wound bed readiness needed to support evidence-based decision making regarding wound care and treatment options, with the potential to save >$7660 in costs associated with failed CTP application over a 4-week period.
Diagnosis and treatment of the invasive extension of bacteria (cellulitis) from chronic wounds utilizing point-of-care fluorescence imaging **Andersen, C.A. 2022 [50]**	**USA**		Typical course of treatment for cellulitis often includes intravenous antibiotics and a hospital stay of several days. Fluorescence extending outside the wound region suggests cellulitis, supporting the decision to initiate oral antibiotics. Use of FLI to detect and manage wound-related cellulitis can mitigate progression of infection and avoid further IV antibiotic courses and high costs associated with inpatient hospital admission.
